# Demonstration of Synaptic Behaviors and Resistive Switching Characterizations by Proton Exchange Reactions in Silicon Oxide

**DOI:** 10.1038/srep21268

**Published:** 2016-02-16

**Authors:** Yao-Feng Chang, Burt Fowler, Ying-Chen Chen, Fei Zhou, Chih-Hung Pan, Ting-Chang Chang, Jack C. Lee

**Affiliations:** 1 Microelectronics Research Center, the University of Texas at Austin, Austin TX 78758, USA; 2 Department of Physics, National Sun Yat-Sen University, Kaohsiung 804, Taiwan

## Abstract

We realize a device with biological synaptic behaviors by integrating silicon oxide (SiO_x_) resistive switching memory with Si diodes. Minimal synaptic power consumption due to sneak-path current is achieved and the capability for spike-induced synaptic behaviors is demonstrated, representing critical milestones for the use of SiO_2_–based materials in future neuromorphic computing applications. Biological synaptic behaviors such as long-term potentiation (LTP), long-term depression (LTD) and spike-timing dependent plasticity (STDP) are demonstrated systematically using a comprehensive analysis of spike-induced waveforms, and represent interesting potential applications for SiO_x_-based resistive switching materials. The resistive switching SET transition is modeled as hydrogen (proton) release from (SiH)_2_ to generate the hydrogen bridge defect, and the RESET transition is modeled as an electrochemical reaction (proton capture) that re-forms (SiH)_2_. The experimental results suggest a simple, robust approach to realize programmable neuromorphic chips compatible with large-scale CMOS manufacturing technology.

In recent years, resistive random access memory (ReRAM) has drawn much interest as a promising candidate for next generation nonvolatile memory (NVM) due to its potential scalability beyond 10 nm feature size using a crossbar structure, fast switching speed, low operating power, and good reliability^1^,^2^,^3^. Traditional charge-based NVM typically includes a charge “trapping layer” within a transistor configuration that requires a high thermal budget and large footprint (typically 6F^2^, where F = minimum feature size)^4^,^5^. Resistive switching (RS) memory operates by controlling device resistance with an external electrical manipulation^6^,^7^,^8^,^9^, leading to better electrical performance, smaller design area (4F^2^), and excellent cycling endurance^10^. Based on the 2013 International Technology Roadmap for Semiconductors (ITRS), ReRAM is one of two recommended candidate technologies for emerging memory devices^11^. Also, resistive-based memories represent a new class of devices compatible with applications that go beyond traditional electronics configurations, for example, three-dimensional (3D) stacking, nano-batteries, neuro-electronics and Boolean logic operations^12^,^13^,^14^,^15^,^16^,^17^.

Neuro-electronics and synaptic electronics are interesting applications for ReRAM that aim to build artificial synaptic devices that emulate the computations performed by biological synapses^15^,^18^. These emerging fields of research potentially have better efficiency in solving complex problems and outperform real-time processing of unstructured data than conventional von Neumann computational systems^19^. There have been many studies of binary metal oxide-based and perovskite oxide-based resistance switching characteristics for synapse-like electronic device development^20^,^21^, which can have operating instability issues due to difficulty in controlling stoichiometric compositions^22^,^23^. Therefore, a simple process that is compatible with conventional complementary metal-oxide semiconductor (CMOS) fabrication allows multi-layer compositional engineering and provides good electrical stability and high yield, which are critical requirements for neuro-electronics realization^24^. Silicon oxide (SiO_x_) has long been used as gate dielectrics for metal-oxide-semiconductor field-effect transistors. In addition to excellent insulating properties, resistive switching properties have been observed in SiO_x_ materials as early as 1962 by Hickmott and 1967 by J. G. Simmons and R. R. Verderber, with additional modeling being done by G. Dearnaley in the 1970s^25^,^26^,^27^. They observed that a simple metal-insulator-metal structure (e.g. Au/SiO_x_/Al, MIM) can form an active memory device based on its repeatable negative resistance phenomenon. Recently, Yao *et al.* have reported SiO_x_-based resistive switching behaviors in vacuum, indicating that this traditionally passive material can be converted to an active memory element and controlled by external electrical activation^28^,^29^,^30^. Several recent reports describe using SiO_2_ as the active switching medium in resistive switching memory devices^31^,^32^,^33^,^34^. We have further demonstrated a Si diode (1D) with low reverse-bias current integrated with a SiO_x_-based memory element (1R) using nano-sphere lithography and deep Si etching to pattern a P^++^/N^+^/N^++^epitaxial Si wafer^35^. The above achievements for intrinsic SiO_x_-based ReRAM indicate: 1) High device yield, forming-free operation, reduced operating voltage, excellent scalability (to dimensions<40 nm in 1D-1R architectures without scarifying the device performance, such as the retention of multilevel states and endurance reliability) and good device stability; 2) Pulsed programming in the 50 ns-regime and low reverse current with large rectification ratio to meet low-energy consumption criteria (>10^6^ for high-conductance states) for integrated 1D-1R nano-pillar architectures; and 3) wide programming resistance dynamic range (potentially up to 10^8^), multi-level states, and excellent reliability. However, the resistive switching mechanisms in SiO_x_ are not well understood and use as an electronic synaptic device has not previously been demonstrated.

In this work, SiO_x_-based resistive switching memory elements (1R) are integrated with Si diodes (1D) using conventional CMOS processing to demonstrate a 1D-1R device with synaptic behaviors. Compared with our previous work (in most cases investigating only the 1R device system), the Si diode provides low reverse-bias current and high power efficiency for future neuromorphic computing array architectures. Unlike other binary or complex metal oxide materials^36^, SiO_x_ has been used in CMOS manufacturing for over 50 years due to its excellent electrical isolation properties, low-cost, high chemical stability, compatibility with mainstream integrated circuit materials, high-throughput processing and large-area production using chemical vapor deposition (CVD). A 1D-1R architecture fabricated at the wafer-scale using conventional CMOS processing can therefore be well-controlled in thickness, size, and electrical characteristics by precisely controlling the doping levels of the diode layers and the temperature and flow-rate of the oxide CVD process^37^. Synaptic device performance is characterized in a prototype 1D-1R array configuration. Robust biological synaptic behaviors such as long-term potentiation (LTP), long-term depression (LTD) and spike-timing dependent plasticity (STDP) are demonstrated with excellent uniformity, low operational variability and good suppression of static power consumption^36^. A bio-inspired proton exchange resistive switching model is used to help characterize this novel application for SiO_x_ materials. The SET transition in the resistive switching memory is modeled as hydrogen (proton) release from the (Si-H)_2_ defect to generate a conductive hydrogen bridge, and the RESET transition is modeled as an electrochemical reaction (proton capture) that re-forms non-conductive (SiH)_2_. The synaptic behaviors exhibited by the 1D-1R device demonstrates good potential for using a simple and robust approach for large-scale integration of programmable neuromorphic chips using CMOS technology.

## Method and Experiment

**Figure 1:**
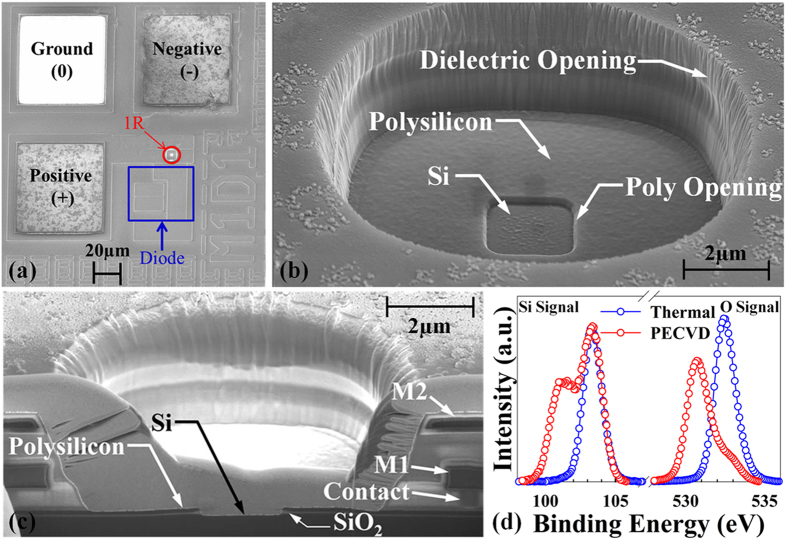
(**a**) Top-down SEM image of 1D-1R architecture. The 1R (red) is adjacent to the 1D (blue) structure. The ground pad (0) is used to bias the substrate, the positive (+) and negative (−) terminals are for applying voltage to the 1D-1R device. (**b**) Tilted top-down SEM image of resistive memory device. (**c**) SEM cross-section image showing metal contact to polysilicon top electrode, metal 1 (M1) and metal 2 (M2) layers, and polysilicon/SiO_2_/Si 1R device. (**d**) Si-2p_2/3_ and O-1s XPS spectra for PECVD oxide and thermal oxide.

Secondary electron microscopy (SEM) images show a top-down view of a 1D-1R test structure (Fig. 1a), a tilted (45°) view of the 1R device (Fig. 1b) and a cross-section image of the 1R device showing layer information (Fig. 1c). The devices were fabricated at XFAB in Lubbock TX using the XC06 CMOS process technology. The 1R device was fabricated by first implanting the Si substrate to form an n-type lower electrode. The active SiO_x_ memory layer was then deposited to a thickness of 40 nm using plasma-enhanced chemical vapor deposition (PECVD). This thickness is known to provide high electroforming yield and good memory endurance^38^. An n-type polysilicon layer was deposited onto the SiO_x_ layer to form the top electrode. An opening in the polysilicon layer was made after all thermal oxidation and implant anneal steps are complete (Fig. 1b). A first dielectric layer was then deposited over the polysilicon top electrode. Tungsten plugs were used to make electrical contact to the n-type Si lower electrode and the polysilicon top electrode. After all the back-end dielectrics and a passivation layer were deposited, the back-end dielectric layers were removed using reactive ion etch (RIE) to the Si substrate. This RIE step cleared-out the SiO_x_ layer inside the hole, and created a SiO_x_ sidewall where the memory device is formed (Fig. 1c). Polymer residue that remained after the post-RIE cleaning steps was removed by a 30-second buffered oxide etch (BOE). The pn diode used in the 1D-1R test structures was formed by an implanted p-well inside a deep n-well with 40 V reverse-bias breakdown voltage, 1 nA reverse-bias leakage current and 0.5 V forward voltage. The active memory area of the 1R device is 2 × 2 μm^2^ and the overall size including metal interconnects is 21.9 × 21.9 μm^2^. The overall size of the 1D device is 41 × 19 μm^2^. A Lake Shore Cryotronics vacuum probe chamber (<1 mTorr) and Agilent B1500A device analyzer were used to electroform devices and measure the DC/AC *I-V* response. The SET process programs the device to a conductive, low-resistance state (LRS). The RESET process programs each device to a low-conductance, high-resistance state (HRS). A Kratos Axis Ultra HSA X-ray Photoelectron Spectrometer (XPS) equipped with a monochromatized aluminum x-ray source was used to analyze several SiO_x_ materials deposited in our laboratory using different methods. Calibration of the binding energy scale was set by fixing the C-(C,H) peak at 284.4 eV. Fig. 1d shows XPS analysis results for the O-1s and Si-2p binding energies in thermal oxide grown by low-pressure chemical vapor deposition (LPCVD) and PECVD oxide. The existence of stoichiometric SiO_2_ can be observed in thermal oxide (binding energy Si: 103.2 eV; O: 532.5 eV) with essentially no sub-oxide bonding being detected. In contrast, the PECVD oxide has non-stoichiometric SiO_x_ (x is about 1.6 based on the peak position and orbital valence) composition in the switching layer, as indicated by the peak binding energies in the XPS spectra (O: 530.5 eV; Si: 101.9 eV and 100.9 eV)^39^,^40^, which may promote low-energy defect generation during the electroforming process.

## Results and discussions

**Figure 2:**
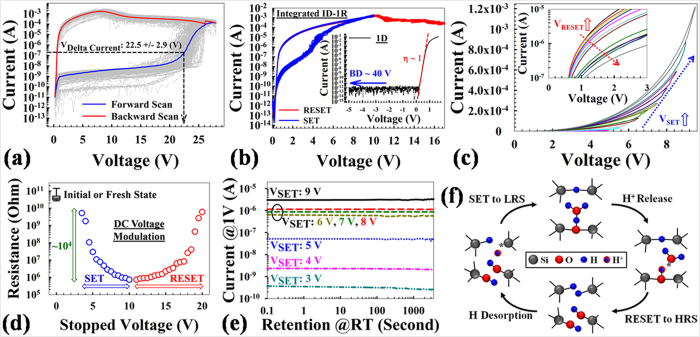
DC sweep resistive switching behaviors of 1D-1R architecture: (**a**) Forward/backward voltage sweeps during electroforming process averaged for 256 devices in a 16 × 16 array (grey curves). The electroforming voltage (V_Delta Current_) is defined as the voltage where maximum current change occurs during the forward sweep. (**b**) 10 I–V resistive switching SET/RESET cycles. The inset shows the average of 100 measurement cycles of diode I-V behavior. (**c**) Effects of voltage modulation on I-V curves in SET process plotted on linear-scale, where the applied SET voltage sweep increases from 3.5 V to 9.5 V in 0.5 V steps. The inset shows effects of voltage modulation on I-V curves in RESET process plotted on log-scale, where the applied RESET voltage sweep increases from 11.0 V to 18.0 V in 0.5 V steps. (**d**) The resistance states of initial fresh device, SET DC voltage modulation, and RESET DC voltage modulation. For SET voltage sweep, increases from 3.5 V to 10 V in 0.5 V steps; for RESET voltage sweep, increases from 11 V to 20 V in 0.5 V steps. The resistance reads at 1V for each state. (**e**) Retention measurement results of multi-state programming obtained by controlling the SET voltage. (**f**) Proton exchange induced resistive switching model and defect transitions. ON state (top) contains Si-H-Si and Si_2_ = O-H_3_O^+^. Electron injection into H_3_O^+^ releases a proton (right-side intermediate state). Electrochemical reaction between the proton (H^+^) and Si-H-Si forms (SiH)_2_ and water absorption forms (SiOH)_2_ to switch the complex OFF (bottom). Charging (SiH)_2_ positive leads to H^+^ desorption from SiH (left-side intermediate state). Proton uptake by absorbed water forms Si_2_ = O-H_3_O^+^ and switches the complex ON (top).

Figure 2a–d shows *I-V* characteristics for DC voltage sweeps applied to the SiO_x_-based 1D-1R devices fabricated by the conventional CMOS process. Voltage was applied to the 1D top electrode (p-type Si) with bottom 1R electrode (n-type Si) at ground. All testing was done in vacuum. To establish reversible resistive switching in each SiO_x_-based 1R ReRAM device, a forward/backward voltage sweep (Fig. 2a) was used to electroform each device, where current is observed to increase dramatically at 22.5 + /− 2.9 V during the forward voltage sweep. Electroforming is completed during the backward voltage sweep from the maximum sweeping voltage to 0 V, resulting in the formation of a conductive filament (CF) and setting the device to a LRS. After electroformation, resistive switching performance of 1D-1R is stabilized by cycling the device multiple times using voltage sweeps (Fig. 2b). The SET process is a 10 V forward/backward sweep without any compliance current limit (CCL) to program the device to the LRS. The RESET process is done by sweeping the voltage to 17 V, where current decreases as the voltage is swept from about 10 V to 17 V; and the device is programmed into a HRS. The HRS/LRS resistance ratio is at least ~10^3^ at 1 V bias, which satisfies sensing requirements^3^,^41^. For diode characteristics, the forward current can reach 100 mA at 2 V (current density 1.15 × 10^−5^ A/μm^2^ at 1V), which indicates a forward current level high enough to support the RESET process. The reverse current is below 1 × 10^−12^ A at −5 V. Compared with Schottky diodes (potentially useful for 3D arrays), the advantages of Si-based PN diodes include low reverse-current, high reverse-bias breakdown voltage, and fewer stability issues^30^. The quality of the Si-based PN diode can dramatically affect diode reverse or forward current characteristics, as well as power consumption (describe below). Also, the chosen Si-based PN diode configuration has high reverse breakdown voltage (>40 V), which is important for SiO_x_-based ReRAM operating in an array. Figure 2c demonstrates the gradual change of resistive states by modulating the voltage sweep range continuously during the SET and RESET (inset) process, respectively. Specifically, SET and RESET voltages were changed from 3.5 V to 9.5 V in 0.5 V increments and from 11 V to 18 V in 0.5 V decrements, respectively, thus potentially enabling multilevel programming in a single memory cell and demonstrate the status stability before/after sweeps. It may be noted that the electroforming voltages measured here (~28 V) are somewhat higher than those measured in previous work on metal-oxide-semiconductor device architectures or nano-pillar type 1D-1R atchitectures^35^,^41^,^42^, which may be due to fewer electrically-active defects being near the SiO_x_ sidewall as a result of the fabrication process. For example, several high temperature steps (>650 °C) were done after PECVD SiO_2_ deposition, namely: polysilicon deposition, thermal oxidation, and implant anneals, which might densify the SiO_2_ layer, reduce the as-deposited defect levels, increase the soft breakdown threshold, and thus increase the filament formation energy during the subsequent electroforming process (resulting in forming voltage increase). Interestingly, the RESET voltage (the voltage at which LRS current begins to decrease) has been found to be greater than or equal to the SET voltage (where HRS current increases sharply), which is a unique characteristic of the SiO_x_-based ReRAM as compared to other materials systems^21^,^43^. The difference between RESET and SET voltages can potentially be controlled by optimizing the series resistance in the circuit, choice of electrode materials, and by doping effects that modulate the interfacial contact resistance^44^. The switching voltage is largely independent of device size and SiO_x_ thickness. Figure 2e shows multilevel retention performance of SiO_x_-based 1D-1R devices obtained by controlling the maximum SET voltage from 3 V to 9 V. The readout current of LRS and HRS is measured at 1 V every 60 seconds after each programming operation. Although the state’s stability still needs to be improved (no equal split of resistance states), the retention reliability test demonstrates multilevel operation by using different SET voltages, and no degradation is observed for more than 10^3^ sec, thus confirming the stable, nonvolatile nature of the SiO_x_-based 1D-1R devices. In recent studies, a possible proton-exchange model consistent with the observed resistive switching *I-V* response has been proposed, as shown in Fig. 2f. 44,45, Several studies have used transmission electron microscopy (TEM) to document the presence of Si nanocrystals within the CF^28^,^46^,^47^, but it is not yet clear whether resistive switching (RS) is the result of an overall increase in nanocrystal size or whether switching occurs in “GAP” regions in between nanocrystals. Most models of ReRAM switching involve the drift or diffusion of O^2−^ ions (or oxygen vacancy defects)^24^, but these models cannot explain the unconventional *I-V* response. For example, the backward scan effect (see Fig. 2a, backward scan) is very difficult to explain using a simple oxygen vacancy switching model. The backward scan effect is a phenomenon where the duration of the reverse sweep during electroforming or RESET determines whether a state change occurs, and has been characterized using DC and AC pulse response in a previous study investigating our resistive switching model^41^. In addition, ambient effects on resistive switching suggest that the defects responsible for switching are hydrogen-passivated or are in some way protected from direct reaction with ambient oxygen and water until a switching events occurs (see Supporting Information, S1, for detailed ambient effect results)^42^,^48^. The detailed interactions between ambient gases and proton (or cation) mobility is an important topic that may provide a deeper understanding of resistive switching mechanisms^49^,^50^,^51^,^52^,^53^, specifically those in oxide-based valence change memory (VCM)-type ReRAMs^54^,^55^,^56^. The models used here to describe the possible SiO_x_-based RS mechanisms differ from most conventional models by considering that the defects responsible for RS may remain localized within the switching region so that resistive switching occurs when a collection of defects are driven between conductive and non-conductive forms^42^. A thorough review of the reported electrical and structural properties of known SiO_x_ defects has identified a plausible model for the conductive filament that is similar to models used to describe stress-induced leakage current and breakdown in SiO_x_ materials, where defect concentration increases as a result of electrical stress to the point where percolation pathways capable of conducting appreciable current (>1 uA) are formed^44^. Incorporating known proton exchange reactions that can dramatically alter the conductivity of specific defects further leads to a model where the LRS has a large concentration of conductive defects within the switching region, and, conversely, when the device is programmed to the HRS, most of the defects are converted to their non-conductive form. The electrically-conductive hydrogen bridge (Si-H-Si) is viewed as the most likely defect responsible for the LRS due to the location of its energy levels relative to the oxide conduction band and its small effective bandgap energy^44^,^45^. Adding a proton to Si-H-Si forms the non-conductive (SiH)_2_ defect and proton desorption from (SiH)_2_ re-forms Si-H-Si, which are well-understood electrochemical reactions that could enable localized switching without incorporating ion diffusion or drift mechanisms into the model. The SET transition voltage from HRS to LRS occurs at ~2.5 V in the I–V response, and is very near the activation energy for proton desorption from SiH (~2.5 eV), thus making the defect transformation from (SiH)_2_ to Si-H-Si a logical assignment for the SET transition^44^,^45^. In this model, the proton that is lost from (SiH)_2_ reacts electrochemically with (SiOH)_2_, which is simply chemisorbed H_2_O, to form the fixed positive charged H_3_O^+^ defect. The transition from LRS to HRS is modeled as being initiated by electron injection into H_3_O^+^ that induces proton release and electrochemical reaction with Si-H-Si to re-form (SiH)_2_^44^,^45^. The localized proton exchange switching model can thus be written as (SiH)_2_ + (SiOH)_2_ ↔ Si-H-Si + Si_2_ = O-H_3_O^+^, where a voltage drop of ~2.5 V across the switching is required to drive the reversible reaction. The RS model not only provides insights into multilevel operational characteristics but also implies a possible biomimetic chemical reaction similar to reactive oxygen species (ROS–like) production for future device characterizations^57^.

**Figure 3:**
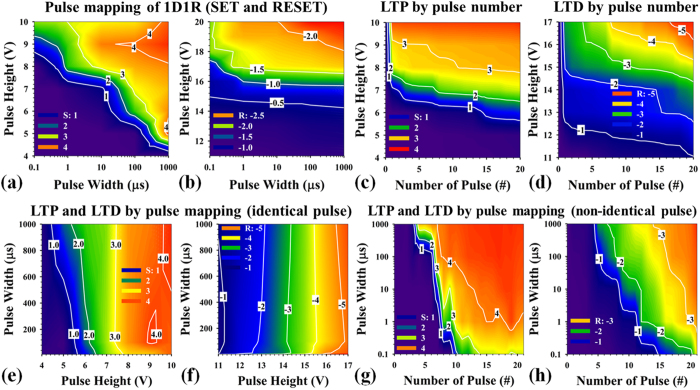
AC pulse mapping contour plots of current-change ratio by modulating pulse height and pulse width to demonstrate synaptic behaviors in 1D-1R architectures: (**a**) SET (S) and (**b**) RESET (R) mapping results of 1D-1R device. (**c**) and (**e**) Long-term potentiation (LTP) and (**d**) and **(f**) long-term depression (LTD) using the identical pulse method as a function of pulse width. For the identical pulse method, pulse height and pulse width are fixed. The mapping procedure is similar to that of Figure S1a. For LTP, the pulse height modulation changes from 11 V to 17 V in 0.3 V increments for each loop, and pulse widths are fixed at 10 μs. The mapping results of using the identical pulse method for LTP are show in (**e**). By selection of final states (after 20 pulses), the conductance change is highly-dependent on the pulse height. For LTD, the pulse height modulation changes from 4 V to 10 V in 0.3 V increments for each loop, and pulse widths are fixed at 10 μs. The mapping results (**f**) are similar and the conductance change for LTD is also highly-dependent on the pulse height rather than pulse width. (**g**) and (**h**) show the LTP and LTD using the non-identical pulse method as a function of pulse width, respectively. For the non-identical pulse method, pulse height modulation changes continuously from 4 V to 10 V in 0.3 V increments (for a total of 21 steps) for LTP, and changes continuously from 11 V to 17 V in 0.3 V increments (for a total of 21 steps) for LTD. The initial states for LTP and LTD mapping are determined by fixed DC conditions: a 17 V single-sweep for HRS and a 10V double-sweep for LRS, respectively. “S” and “R” denote the increment/decrement of current state changes after applying the AC pulse (defined as Log_10_ (I_n_/I_nitial_), where I_n_/I_Initial_ is current ratio measured at 1 V after/before the pulse is applied).

Figure 3a–h show contour plots of the current-change ratio achieved by modulating the AC pulse height and pulse width applied to 1D-1R devices for both SET and RESET switching events, leading to optimized waveform designs for a biological synaptic device. The current-change ratio is defined as log_10_ (I_FINAL_/I_INITIAL_), where I_INITIAL_ and I_FINAL_ are the currents measured at 1 V before and after applying the programing waveform, respectively. The SET switching events (S) increase current through the device, leading to positive current change ratios, whereas RESET switching events (R) decrease device current and lead to negative current change ratios. The pulse mappings are generated using the Agilent B1500A device analyzer in a three-step process: (1) Initial states are programmed using a fixed DC voltage before the pulse waveform is applied (S2, for the detailed state mapping procedure); (2) The pulse waveform is applied; and (3) Device state is read by measuring the current at 1 V before and after each pulsed switching event. The SET/RESET sweeps from same initial resistance state is to eliminate the accumulating SET/RESET effect after each cycle. One can observe by inspecting the contour lines in Fig. 3 that when larger pulse heights (higher voltages) are applied to the device, shorter pulse widths are needed to achieve a similar current-change ratio. In general, we find that a single 1R device operates at higher speed and requires lower programming voltages as compared to a 1D-1R device. The higher operating voltages and lower operating speed of the integrated 1D-1R device may result from higher parasitic resistance in the Si electrodes, their contacts and the diode, as well as higher parasitic capacitance in the diode, all of which can act to degrade the pulse mapping results shown in Fig. 3 (a,b). It should be noted that current sneak-path issues in arrays and writing disturbance of 1R devices would cause misread problems and state disturbance, and substantially increase standby power consumption and information instability. The 1D-1R devices are used to suppress sneak-path currents, and perform much better than 1R devices in an array architecture (potential 1Gbit array support in 10% readout-margin at 1V read). From Fig. 3a,b, it can be calculated that the switching energies to achieve at least a one-order-of-magnitude change in resistance in the 1D-1R architecture are about 0.01 pJ for SET and 1.54 nJ for RESET operations. However, due to the suppression of sneak-path current, the standby power during a 1 V read operation can be dramatically reduced in 1D-1R devices (1 pW) as compared to 1R devices (1 μW, due to 1R nonpolar switching behaviors)^58^. Minimizing the total power consumption due to sneak-path current is as crucial as reducing the synaptic dissipation.

Most importantly, the pulse mapping results not only demonstrate the potential for multilevel programming by properly designing the pulse waveforms for SET and RESET operations, but also demonstrate the potential to realize biological synaptic behaviors. Figure 3c–h demonstrate the optimization waveform design for biological synaptic behaviors in 1D-1R SiO_x_-based resistive switching memories. The long-term potentiation (LTP)/long-term depression (LTD) are a long-lasting enhancement/reduction in signal transmission between two neurons (similar with long-lasting conductance increase/decrease between HRS and LRS for resistive-type memory devices), which can be realized by designing the SET and RESET pulse waveform to use either identical (fixed pulse width and pulse height, as shown in Fig. 3c–f and discussed further in Supporting Information, S3) or non-identical (variable pulse width or pulse height, as shown in Fig. 3g,h) pulsing methods. Both methods can be used to demonstrate a SiO_x_-based synaptic device or be adapted to a large number of emerging memory devices. It may be noted that when the dynamic range was evaluated in detail and the trade-offs between high dynamic range and gradual multilevel programming performance (Fig. 3e–h) were considered, it was found that non-identical pulse waveform methods may have certain advantages. (Dynamic range is defined as the maximum achievable resistance of the HRS divided by the minimum resistance of the LRS.) Although non-identical pulsing might require a more complex neuromorphic circuit, our results show that this approach enables more efficient programming to target states while maintaining a larger dynamic range (Fig. 3g,h). The use of non-identical pulse heights ranging from 4 V to 10 V in 0.3 V increments (for LTP) and ranging from 11 V to 17 V in 0.3 V decrements (for LTD) allow the dynamic range to be mapped for pulse widths ranging from 100 ns to 1 ms, thereby realizing biological synapse behaviors in the SiO_x_-based 1D-1R architecture (Fig. 3g,h). The switching energy is defined as I × V × δt, where δt is the pulse width. For δt = 100 ns, the smallest switching energies are ~6 fJ and ~130 pJ for LTP and LTD, respectively. The larger energy for LTD is mainly due to the lower resistance of the LRS (~93 kΩ) compared to the HRS (~260 MΩ), which results in higher switching current (118.28 μA) for the RESET process than for the SET process (15.38 nA). In order to minimize synaptic energy consumption all three components—programming current (~nA level switching), pulse amplitude (<1 V) and programming time (<10 ns)—need to be minimized. In SiO_x_-based ReRAM and in other material systems, an exponential voltage–time relationship is commonly observed. A small increase in programming voltage will decrease programming time exponentially, as shown in Fig. 3(a) and Figure S2 (c). For RESET process (both 1R and 1D-1R structures, Fig. 3(b) and Figure S2 (d)), the process integration may result in certain level of distortion (parasitic resistance/capacitance and possible parasitic depletion region capacitance from 1D) to affect the pulse mapping results. Hence, low programming energy is obtained by minimizing the programming time (traded off by increasing the pulse amplitude slightly) for ReRAM. Further decreases in synaptic energy consumption during the switching process to fJ levels will be challenging but important to build very large-scale systems (the designed pulse waveform optimization and generation is in process).

**Figure 4:**
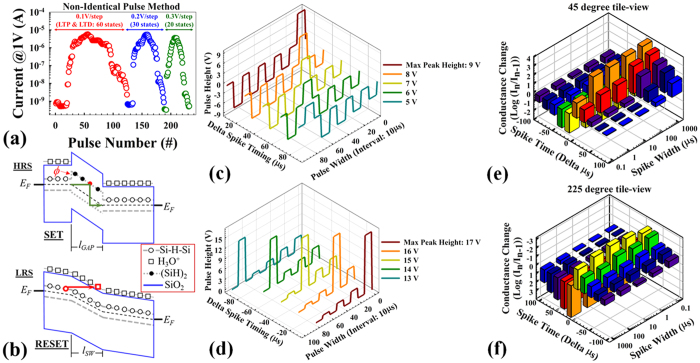
Demonstration of a SiO_x_–based synaptic device. (**a**) Sequential LTP/LTD behaviors as a function of increment/decrement voltage steps (0.1 V, 0.2 V, and 0.3 V) by non-identical pulse form. For the non-identical pulse method, pulse height modulation changes continuously from 4 V to 10 V for LTP, and changes continuously from 11 V to 17 V for LTD. Pulse width is fixed at 10 μs in both cases. (**b**) Energy band diagrams: For HRS and SET process, showing theoretical bandgap of (SiH)_2_ defect within gap region of length *l*_*GAP*_, theoretical bandgap of Si-H-Si defects outside the gap region, and trap-assisted-tunneling SET transition (green arrow). Barrier height to electron transport is *ϕ* ~ 0.8 eV. For the LRS and RESET process, showing theoretical bandgap of Si-H-Si, H_3_O^+^ energy level, switching region of length *l*_*SW*_, and Fowler-Nordheim tunneling RESET transition (red arrow). (**c–d**) A pulse waveform design using the non-identical pulse method for demonstration of spike-timing-dependent plasticity (STDP) as a function of spike pulse width intervals. For the potentiation of conductance strength change, the overall pulse waveform (pulse width fixed at 10 μs in this case) based on the delay of spike timing between neurons is shown in (**c**). Similarly, for the depression of conductance strength change, the overall pulse waveform (pulse width fixed at 10 μs in this case) based on the delay of spike timing between neurons is shown in (**d**). (**e,f**) A demonstration of spike-timing-dependent plasticity (STDP) using the non-identical pulse method with different spike widths. Each colored bar shows the average of 3~5 measurements. (**e**) Emphasizes potentiation direction of STDP with positive delta time (45 degree tilted). (**f**) Emphasizes depression direction of STDP with negative delta time (225 degree tilted). The definition of conductance change is as Log_10_ (I_n_/I_nitial_), where I_n_/I_Initial_ is current ratio measured at 1 V after/before the pulse is applied.

Such flexible artificial control built with synaptic devices could provide a suitable platform for a broad range of computing applications, as shown Fig. 4. Some of the advantages that SiO_x_-based synaptic devices provide over other resistive switching materials include a higher dynamic range (~10^4^)^41^ and the potential to achieve as many as 10–60 multi-level states (depend on the stability) in both LTP and LTD by changing the increment/decrement of the voltage step, as shown in Fig. 4a. (Supporting Information Figure S4 shows LTP and LTD realized in 1R architecture and 1D-1R LTP and LTD endurance.) These advantages may arise as the result of there being a large number of defects within the switching region of the memory device. Switching is modeled as a change in conductivity of a group of defects within the switching region. In this framework, defects are not created or destroyed, but are simply driven between conductive and non-conductive forms by proton exchange reactions that are known to occur in SiO_x_ materials (Fig. 2f)^45^. The SET and RESET switching transitions can be described in more detail with the aid of the electron energy band diagrams shown in Fig. 4b, which were constructed using the thermodynamic and switching charge-state energy levels reported by Peter Blochl in 2000^59^. The ideal energy band diagrams in Fig. 4b represent only a single electron pathway through the memory device, whereas in reality there are likely many such percolation pathways in parallel. The SET transition is modeled as being the result of trap-assisted electron tunneling through (SiH)_2_ defects (a voltage-triggered mechanism, due to less current flow in the initial stage of SET process) that stimulates H^+^ desorption and reaction of H^+^ with absorbed water (SiOH)_2_ to form conductive Si-H-Si and H_3_O^+^ (Fig. 2f). Trap-assisted tunneling can only occur when the bias across the switching region is ≥2.6 V, which is the effective band gap of the (SiH)_2_ defect (S5) and compares well with the observed minimum SET voltage of ~2.5 V in the I-V response^44^,^45^. The RESET transition is modeled as being the result of Fowler-Nordheim electron tunneling into the H_3_O^+^ defect (possibly current-induced Joule heating due to large current flow through the filament) that stimulates proton release and electrochemical reactions to re-form (SiH)_2_ and (SiOH)_2_ (Fig. 2f)^45^. More detailed explanations of the defect energy levels and effective bandgaps are provided in the Supporting Information (S5). The band diagrams shown in Fig. 4b are found to be consistent with measured electron energy barriers^45^ and electroluminescence results reported for similar devices^47^.

Figure 4c–f demonstrates that the SiO_x_-based 1D-1R architecture can mimic spike-timing-dependent plasticity (STDP), a biological process that adjusts the strength of connections between two neurons in a synapse gap junction region that is an electrically conductive link between the pre- and post-synaptic neurons. Two pulse generator sources are used to simulate the pre- and post-synaptic neurons. This provides the pulse waveforms using the non-identical pulse method (also used in various types of emerging memory devices or materials systems) for demonstration of STDP (S6). By design of pre-neuron and post-neuron spikes in neuromorphic circuits, the strength of the conductance change can be modulated based on the spike timing delta (∆t) between the two neurons (Fig. 4c,d and S6). Figure 4e,f demonstrates a total of 10 different states of STDP biological behavior for depression and potentiation with n = 2, 4, 6, 8, 10 and as a function of spike width modulation, ranging from 100 ns to 1 ms. For example, the depression of conductance change strength can be achieved by using multi-step spike heights from −4 V to 0 V in the pre-neuron state and a single spike height fixed at 13 V in the post-neuron state, with both neurons having a fixed pulse width of 10 μs and a firing period of 20 μs, as shown in Fig 4e,f. When the time delay difference is −10 × (n-1) μs, where n is an even number, the total spike waveform (post-neuron spike minus pre-neuron spike) applied to the synapse gap junction region can adjust the conductance ratio between two neurons over the range from 10^−3^ to 0.1 in the depression direction (RESET process) as compared with the initial LRS conductance (Fig. 4f). Similarly, the potentiation of conductance change strength can be achieved by using multi-step spike heights from 4 V to 8 V in the pre-neuron state and a single spike height also fixed at 13 V in the post-neuron state, with both neurons having a fixed pulse width of 10 μs and a firing period of 20 μs. When the time delay difference is 10 × (n-1) μs, where n is an even number, the total spike waveform (post-neuron spike minus pre-neuron spike) applied to the synapse gap junction region can in this case adjust the conductance ratio between neurons over the range from 10^3^ to 0.01 in the potentiation direction (SET process) as compared with the initial HRS conductance (Fig. 4e). It may be noted that the 1D-1R architecture not only avoids sneak-path issues and lowers standby power consumption, but also helps to realize STDP behaviors. Without the 1D rectification characteristics in reverse-bias polarity, the above spiking forms cannot be implemented due to the unipolar nature of the 1R device, specifically in the potentiation behaviors under negative bias. In the 1R case, an applied voltage above the RESET threshold voltage (for example, −9 V) can trigger the RESET process and induce depression behaviors instead of potentiation behaviors. Also, for depression behaviors, when the time delay difference is smaller than the spiking width, the remaining 4 V spike height in this case would not fire the synapse towards a LRS in the depression direction (see Fig. 3h). Therefore, by carefully designing the firing pulses between neurons in the neuromorphic circuit, a biological synapse behavior can be demonstrated with 1D-1R SiO_x_-based resistive switching memories.

**Figure 5:**
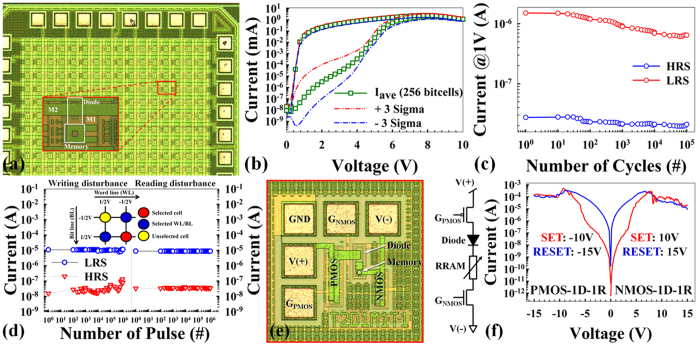
Electrical variation and reliability results for array structure for potential use in future neuromorphic computing applications. (**a**) Optical image of a 16 × 16 bit cell array test chip. (**b**) Averaged data for 256 bit cells, with each bit cell programed using 10 SET/RESET cycles immediately after electroforming, SET: 10 V double-sweep, RESET: 20 V single-sweep. Total number of SET sweeps averaged: 2,560. (**c**) 100k SET-RESET cycles achieved under AC bias conditions (SET: 9 V, 100 ns; RESET: 15 V, 500 ns; READ: 1 V, 1 μs) in 1D-1R architecture. At least 1 order-of-magnitude HRS/LRS ratio was maintained. (**d**) Writing/Reading disturbance of unselected device under worst-case conditions (“1/2 bias” scheme). (**e**) Optical image of a PMOS-1D-1R-NMOS test structure and circuit schematic. The ground (GND) pad provides a substrate bias and reference voltage for transistors. V(+) is the voltage applied to the PMOS transistor (with I-V response shown in (**f**), left panel); G_PMOS_ is the gate bias for the PMOS transistor. V(−) is the voltage applied to the NMOS transistor (with I-V response shown in (**f**), right panel); G_NMOS_ is the gate bias for the NMOS transistor. (**f**) DC sweep resistive switching behaviors of CMOS-1D-1R architecture. The left panel shows the resistive switching results of PMOS-1D-1R architecture. PMOS gate bias is −14 V, applied V(+) is −10 V for SET and −15 V for RESET. The right panel shows the resistive switching results of 1D-1R-NMOS architecture. NMOS gate bias is 1 V, applied V(−) is 10 V for SET and 15 V for RESET.

Figure 5 shows robust electrical reliability and low variation in a 1D-1R-array structure that can potentially be used in future neuromorphic computing applications. Figure 5a shows a portion of a test chip containing a 16 × 16 bit cell array. Each bit cell is comprised of a Si PN diode isolation element and a SiO_2_-based resistive memory element. The 16 × 16 array was fabricated using the same process sequence as described above for the individual 1R and 1D devices. As shown in Fig. 5a, adjacent memory/diode bit cells were connected in a crossbar architecture using metal 1 (M1) lines running vertically and metal 2 (M2) lines running left-to-right in the image. The metal lines run to 32 wire-bond pads positioned around the array perimeter. Four additional wire-bond pads are used for ground connections. The array test chips were sawed from the wafer and wire-bonded into a 64-pin package so that all 36 wire-bond terminals in the array are controlled during electrical testing in a custom-made memory array characterization and test system. Electroforming yield of the 256 bit cells in the array was 98% (aided by the 1D 40 V reverse-bias breakdown voltage). Of these yielding devices, 100% passed a quick, 10-cycle switching performance test without failure. Figure 5b shows the average and ±3-sigma variation of resistive switching behaviors in the 16 × 16 bit cell array cycled using a 10 V double-sweep for SET and 20 V single-sweep for RESET. In this case, the 3-sigma LRS/HRS current ratio at 1 V read bias, was at least 6 × 10^3^. A gradual change in the SET transition is observed over the voltage range from 3.5 V to 6 V, thus allowing programming of the multilevel states that are required for a robust neuromorphic circuit design, and which are accompanied by excellent sub-μs transitions with at least 10× resistance ratio after 10^5^ cycles (Fig. 5c). A 2 × 2 array of integrated 1D-1R bit cells with unipolar programming strategy shows excellent write/read disturbance immunity after 10^6^ pulses for unselected devices and a clear programming window >100 (Fig. 5d). In addition to 1D-1R device arrays, the hybrid CMOS/synaptic device architecture shown in Fig. 5e has been successfully demonstrated as shown in Fig. 5f by the *I-V* resistive switching plots. The 1D-1R architecture with SiO_x_-based resistance switching devices and the structure of artificial neural networks map naturally onto hybrid CMOS/synapse circuits (front-end logic operation and waveform optimization, and back-end 1D-1R neuromorphic functions) that can be designed on a single chip to provide predictable results with an ultimate scaling potential of CMOS technology to the sub-10-nm level, which could possibly challenge the complexity and connectivity of the human brain.

## Summary

In summary, we have demonstrated potentiation, depression and spike timing dependent plasticity in a synaptic device built using a SiO_x_-based 1D-1R architecture. Proton-induced resistive switching behaviors in the SiO_x_ memory element were discussed, where the SET threshold is modeled as proton desorption from the (SiH)_2_ defect to generate the conductive hydrogen bridge, Si-H-Si, and the RESET transition is modeled as proton release and capture to re-form non-conductive (SiH)_2_. The electrical results demonstrate that the technology has good potential for providing a simple and robust approach for large-scale integration of programmable neuromorphic chips using CMOS technology, and represent a critical milestone regarding the potential use of SiO_2_–based resistive memory as a synaptic device in future synthetic biological computing applications.

## Additional Information

**How to cite this article**: Chang, Y.-F. *et al.* Demonstration of Synaptic Behaviors and Resistive Switching Characterizations by Proton Exchange Reactions in Silicon Oxide. *Sci. Rep.* **6**, 21268; doi: 10.1038/srep21268 (2016).

## Supplementary Material

Supplementary Information
