# Supplementary material for: Demonstration of Synaptic Behaviors and Resistive Switching Characterizations by Proton Exchange Reactions in Silicon Oxide
Source: Sci Rep. 2016 Feb 16;6:21268. doi: 10.1038/srep21268 (PMC4754682; doi:10.1038/srep21268)
Supplement: Supplementary Information [file srep21268-s1.pdf]

Support Information for Demonstration of Synaptic Behaviors and Resistive Switching Characterizations by Proton Exchange Reactions in Silicon Oxide

[1,*]*Yao-Feng Chang*, [1]*Burt Fowler*, [1]*Ying-Chen Chen*, [1]*Fei Zhou*, [2]*Chih-Hung Pan*,

[2]*Ting-Chang Chang*, *and* [1]*Jack C. Lee*

[1]*Microelectronics Research Center, the University of Texas at Austin, Austin TX 78758, USA.*

[2]*Department of Physics, National Sun Yat-Sen University, Kaohsiung 804, Taiwan.*

Abstract: We realize a device with biological synaptic behaviors by integrating silicon oxide (SiO_x) resistive switching memory with Si diodes. Minimal synaptic power consumption due to sneak-path current is achieved and the capability for spike-induced synaptic behaviors is demonstrated, representing critical milestones for the use of SiO_2–based materials in future neuromorphic computing applications. Biological synaptic behaviors such as long-term potentiation (LTP), long-term depression (LTD) and spike-timing dependent plasticity (STDP) are demonstrated systematically using a comprehensive analysis of spike-induced waveforms, and represent interesting potential applications for SiO_x-based resistive switching materials. The resistive switching SET transition is modeled as hydrogen (proton) release from $(SiH)_2$ to generate the hydrogen bridge defect, and the RESET transition is modeled as an electrochemical reaction (proton capture) that re-forms $(SiH)_2$. The experimental results suggest a simple, robust approach to realize programmable neuromorphic chips compatible with large-scale CMOS manufacturing technology.

Keywords: Proton exchange, Resistive switching, Synaptic device, Silicon oxide, Neuromorphic Computing.

---

[*] Corresponding Author Email: yfchang@utexas.edu

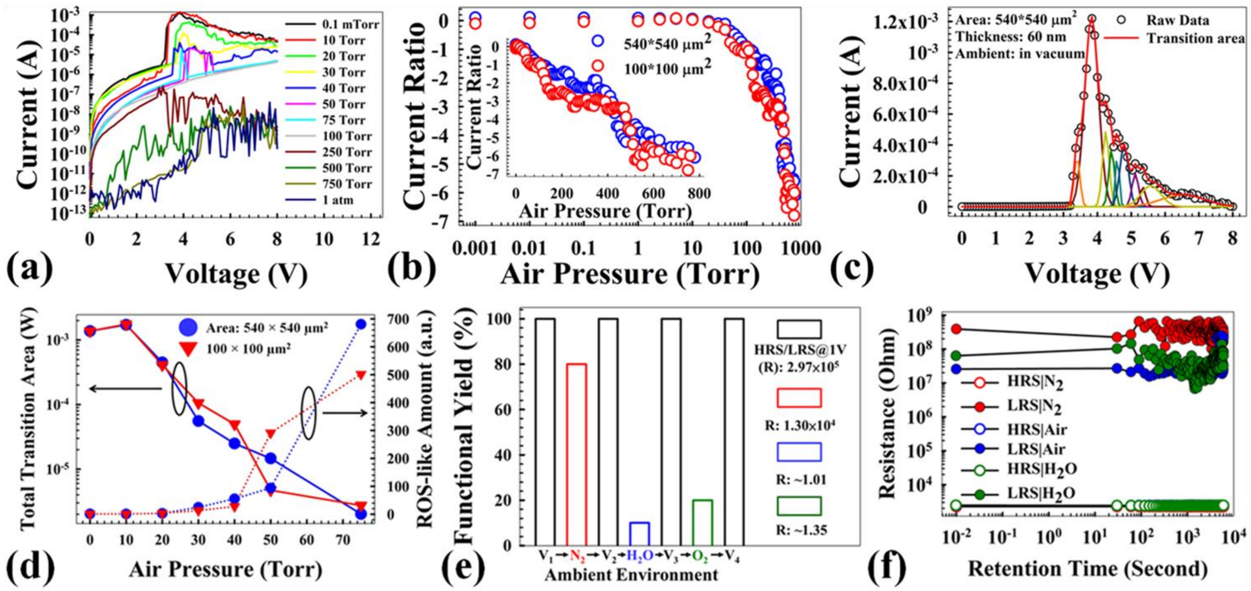

Figure S1. Ambient effects on silicon oxide resistive switching. The device structure is TaN/SiO_2/N^+ Si. The 60 nm SiO_2 layer was deposited by reactive sputtering. After TaN electrode fabrication, an exposed sidewall edge was formed in the SiO_2 layer using wet etching.[53] **(a)** I-V curves of DC voltage sweeps from 0 V to 8 V as a function air pressure (0.1 mTorr – 760 Torr). At 0.1 mTorr and 10 Torr, devices exhibit a peak in current near 4.0 V followed by a continuous drop in current to ~ 8 V, which is a characteristic RESET response. As air pressure increases to > 10 Torr, the I-V plot begins to depart substantially from the characteristic RESET response, showing a reduction of the current peak (20 Torr – 100 Torr), eventually leading to the inability to switch state at 1 atm (760 Torr). This experiment makes it clear that O_2 and/or H_2O vapor in the air are in the immediate vicinity of the conductive filament whenever devices with an unpassivated sidewall edge are exposed to air. **(b)** The current degradation ratio plotted versus air pressure on log scale and linear scale (inset) as a function of device area. The current degradation ratio is defined as Log_{10} (I_n/I_1), where I_n/I_1 is the current measured at 4 V after/before air is introduced into the measurement system. No device area dependence was observed, as would be expected for a conductive filament formed near the SiO_2 sidewall edge.

These results indicate that both the SET transition at ~ 3.0 V and the RESET transition are disabled by either oxygen (O_2) or water (H_2O) in the ambient (see **(e)** for detailed effects of different ambient gases), consistent with the hypothesis that the defects responsible for resistive switching are hydrogen-passivated and are inert to reactions with O_2 and H_2O in the air until the voltage increases to the point where a switching event occurs (see **(f)** for retention properties in different ambient gas environments). **(c)** The transition area of the I-V response was used to characterize the effects of air pressure on device switching. The sample shown has device area of 540×540 μm^2 and is operating in vacuum. The rainbow-colored lines are the fitting peaks used to estimate the area. **(d)** The total transition area (current × voltage = power) and bio-inspired reactive oxygen species mimicking (ROS-like) behavior plotted versus air pressure as a function of device area. Total transition area decreases with increasing air pressure. The ROS-like amount is the total transition area normalized to the 0.1 mTorr condition, and begins to increase when air pressure increases above ~ 20 Torr. **(e)** Resistive switching functional yield over 10 switching cycles as a function of sequentially-applied ambient gas environments. The vacuum environment is inserted between different ambient gases for experimental control. For vacuum conditions, the switching functionality yield is 100 %, and for nitrogen (N_2) environment, the yield is 80 %. The pure H_2O and O_2 environments substantially lower switching yield to 10 % and 20 %, respectively, demonstrating that both O_2 and H_2O strongly attack the conductive filament. The fact that all devices recover when placed back into vacuum demonstrates that the filament is not permanently damaged by O_2 or H_2O. Of note, although memory window (HRS/LRS read @1V, named "R") is degraded in N_2 environment, it is still within the typical values for silicon oxide resistive switching memory. However, for H_2O and O_2 environments, the memory window has essentially disappeared (R ~ 1.01 and 1.35, respectively) and no transition events happen as the

result of electrical stimulation. These experiments strongly suggest that hydrogen passivation is lost during a switching event, which allows O_2 and H_2O to react with defects in the filament, but the reaction can easily be reversed by placing the device back into vacuum. **(f)** The retention properties of resistive states in different ambient gas environments. The LRS and HRS resistance are monitored at 0.2 V after every 30 seconds of time. Data retention is stable for different ambient gases with a resistance ratio of at least three orders of magnitude and no significant degradation observed for more than 6000 sec, further confirming that the defects responsible for device switching remain hydrogen-passivated at low bias and are thus inert to reactions with O_2 and H_2O.

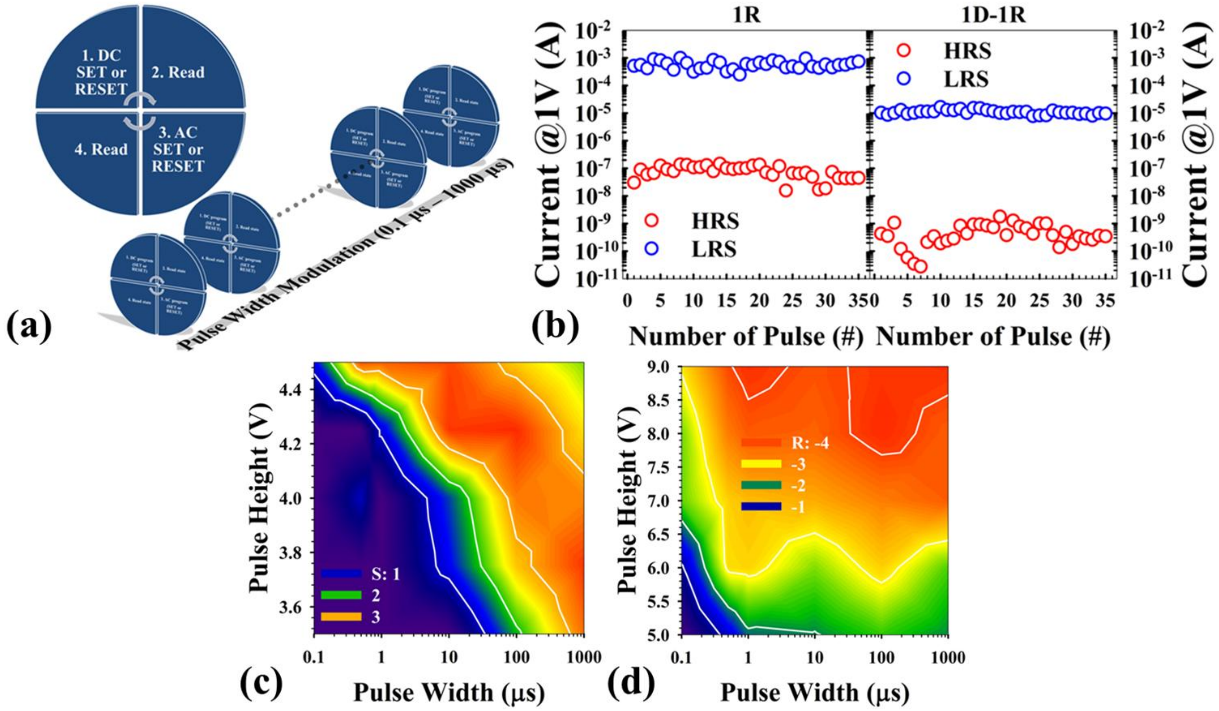

Figure S2. **(a)** The sequential procedures of pulse mapping for device characterizations. Each circulant matrix represents a loop of pulse height mapping. The four steps include: **1)** DC SET/RESET programming to the LRS/HRS; **2)** Reading the state at 1 V DC; **3)** Apply a voltage

waveform with fixed pulse width (i.e. 0.1 μs) and modulate the pulse height for each event (i.e. change from 4 V to 10 V in 0.2 V increments for SET mapping in a 1D-1R architecture, or change from 5 V to 9 V in 0.2 V increments for RESET mapping in a 1R architecture); and **4)** Reading the state at 1 V DC. The number of switching events in each loop is dependent on the design of incremental voltage steps, i.e. when changing from 4 V to 10 V in 0.2 V increments, there are a total of 30 switching events. After finishing a loop of pulse height mapping, the pulse width is incremented, i.e. from 0.1 μs to 0.5 μs, and the next loop of pulse height mapping is then performed. The stability of DC initialized states are confirmed, as shown in **(b)** for 1R and 1D-1R architectures. The DC programming provides a uniform state for subsequent pulse mapping. For the 1R case in **(b)**, the RESET process is implemented using 8 V single-sweeps while in the LRS, and the SET process is implemented using 4 V double-sweeps with 1 mA compliance current (C.C.) limit while in the HRS; in the 1D-1R case, the RESET process is performed using 20 V single-sweeps while in the LRS, and the SET process is done using 10 V double sweeps while in the HRS. (**c-d**) AC pulse mapping contours achieved by modulating pulse height and pulse width: (**c**) SET and (**d**) RESET mapping results of 1R device. The initial states for SET and RESET mapping are conditioned as described above by a fixed 4 V DC double-sweep with 1mA compliance current limit for the LRS, and an 8 V DC single-sweep for HRS. The pulse height ranged from 3.5 V to 4.5 V and pulse width ranged from 100 ns to 1 ms for the SET process. The pulse height ranged from 5.0 V to 9.0 V and pulse width ranged from 100 ns to 1 ms for the RESET process. "S" and "R" denote the SET increment/RESET decrement of current state changes after applying the AC pulse. The current state change is defined as Log_{10} (I_n/I_{nitial}), where $I_n/I_{Initial}$ is the current ratio measured at 1 V after/before the pulse waveform is applied.

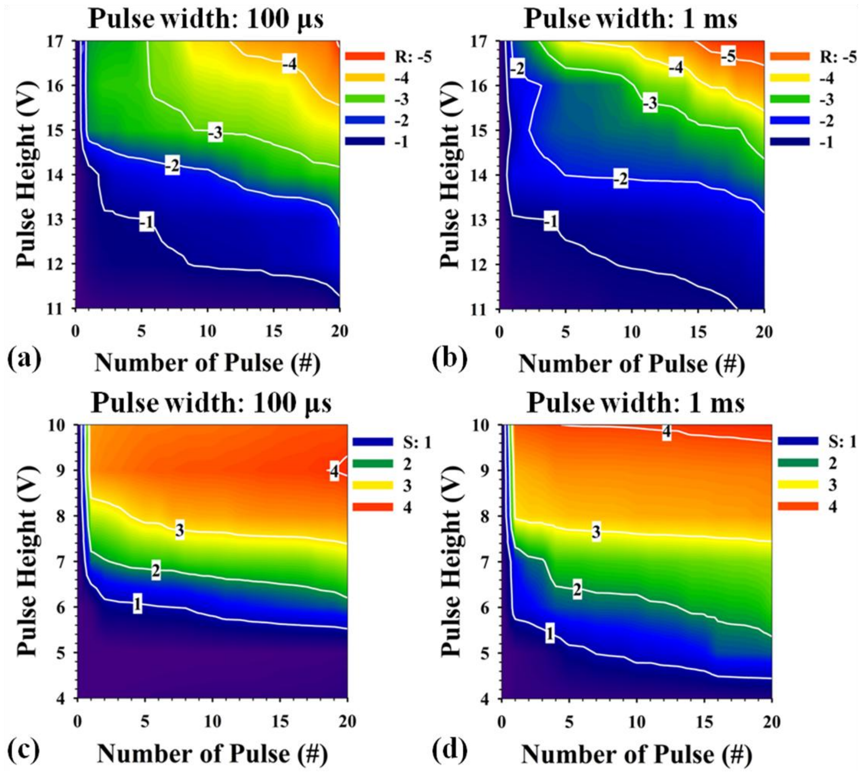

Figure S3. Demonstration of a synaptic device in 1D-1R architecture with SiO_x-based resistive switching memory. (**a-b**) Long-term depression (LTD) and (**c-d**) long-term potentiation (LTP) using the identical pulse method as a function of pulse width. For the identical pulse method, pulse height and pulse width are fixed. The mapping procedure is similar to that of Figure S1a. After DC programming of an initial state, each AC condition is continuously applied 20 times while reading the conductance state change, which is similar to cycling 20 times between step 3 and step 4 in the Figure S1a case. For LTD, the pulse height modulation changes from 11 V to 17 V in 0.3 V increments for each loop, and pulse width changes are (**a**) 100 μs, and (**b**) 1 ms in this case. For LTP, the pulse height modulation changes from 4 V to 10 V in 0.3 V increments for each loop, and pulse width changes are (**c**) 100 μs, and (**d**) 1 ms. “S” and “R” denote the SET increment/RESET decrement of current state changes after applying the AC pulse. The current state change is defined as Log_{10} (I_n/I_{nitial}), where $I_n/I_{Initial}$ is the current ratio measured at 1 V after/before the pulse waveform is applied.

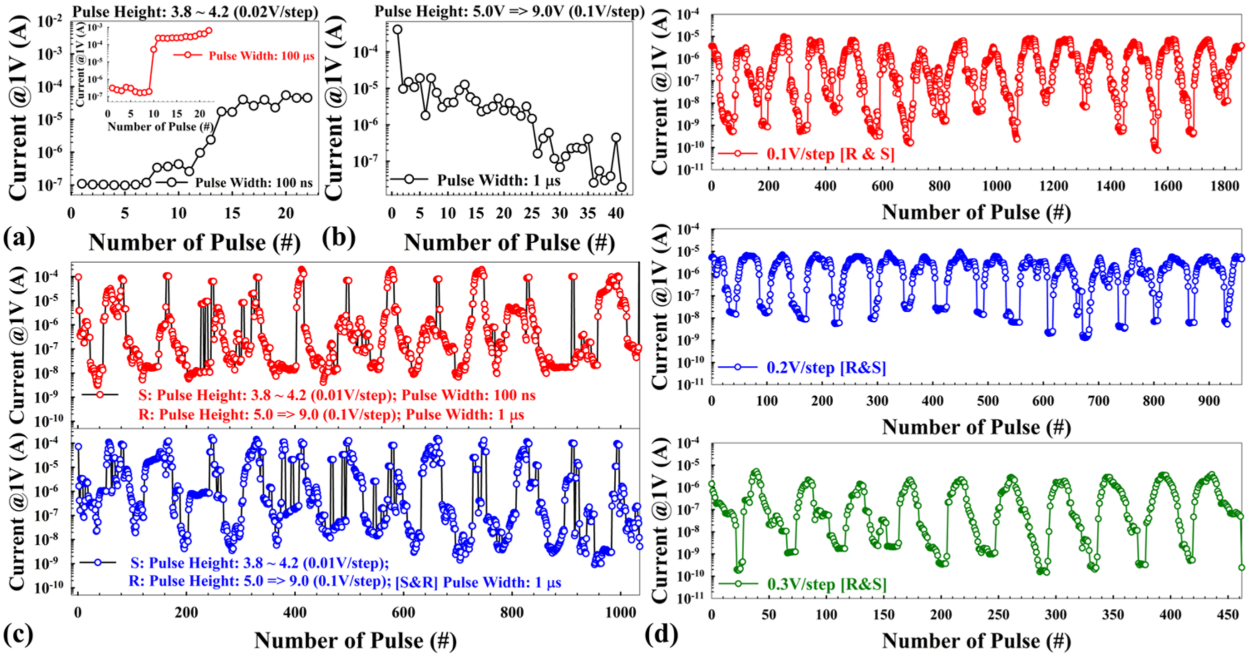

Figure S4. Sequential LTP/LTD behaviors demonstrated using the non-identical pulse method in 1R architecture and the endurance of LTP/LTD in 1D-1R architecture. In (**a**) LTP behavior is demonstrated by changing the pulse height from 3.8 V to 4.2 V in 0.02 V increments: a 100 ns pulse-width cannot realize multilevel states (inset), and a dramatic and sharp state change is observed; however, using 1 μs pulse-widths allows multi-level states to be achieved, leading to a gradual state change as a function of the number of applied pulses. In (**b**) LTD behavior is achieved by changing pulse height from 5.0 V to 9.0 V in 0.1 V increments while using a 1 μs pulse width. (**c**) The endurance of sequential LTP/LTD behaviors using non-identical pulses in 1R architecture with different pulse-widths in potentiation: 100 ns (top) and 1 μs (bottom). (**d**) The endurance of sequential LTP/LTD behaviors using non-identical pulses in 1D-1R architecture with different voltage increment steps for potentiation and depression: 0.1 V (top), 0.2 V (middle), and 0.3 V (bottom). For depression, the pulse height modulation changes from 11 V to 17 V with 10 μs pulse width; for potentiation, the pulse height modulation changes from 4.0 V to 10 V with 10 μs pulse width. The 1D-1R architecture in **(d)** is observed to mimic

biological signals much better than the 1R architecture in **(c)**. The “S” and “R” denote potentiation and depression directions, respectively.

**Defect**	**Charge-States**	**E_C**	**E_{TH}**	**E_V**	**ΔE_G**
$(SiH)_2$	+/0	-1.07	-2.74	-3.67	2.60
Si-H-Si	0/-	1.58	0.74	-0.13	1.71

Figure S5. Defect positive (+), neutral (0) and negative (–) switching charge-states, unoccupied switching charge-state energy levels that form an effective conduction band-edge (E_C), thermodynamic energy levels (E_{TH}), occupied switching charge-state energy levels that form an effective valence band-edge (E_V) and effective bandgap energies (E_G) referenced to the Si midgap energy in units of eV.[66] To determine the relative band offsets in the band diagrams (Fig. 4b), the standard methodology used for semiconductors was employed where the thermodynamic energy level of each defect was aligned to the electrode Fermi levels at 0 V bias, thus pinning the thermodynamic energy levels of the defects to the electrode Fermi level.[65] The ideal band diagrams in Figure 4b of the main text assume electrodes with Fermi levels at the Si midgap energy and mono-energetic defect energy levels. Defect spacing is assumed to be uniform with one defect cluster every 1/2 nm, which is based on the average electron hopping distance determined by fitting the low-voltage I-V response to the hopping conduction expression.[54] In the LRS band diagram shown in Fig. 4b (bottom), the conductive filament (CF) is comprised of conductive Si-H-Si and positive-charged H_3O^+, where Si-H-Si has an effective bandgap of 1.71 eV. Hopping transport through the effective conduction band of Si-H-Si is presumed to be responsible for the relatively high conductivity and small electron energy barrier of ~ 0.1 eV

measured in devices programed to the LRS.[54] The RESET transition is initiated when Fowler-Nordheim tunneling from Si-H-Si into H_3O^+ releases a proton from the H_3O^+ defect. The HRS band diagram shown in Fig. 4b (top) is drawn for the case where all defects in the switching region are converted into non-conductive $(SiH)_2$ and $(SiOH)_2$ to form a conductance gap along the filament. The $(SiH)_2$ defects have an effective bandgap of 2.6 eV (Fig. S4), leading to a discontinuity in the conduction band of ~ 0.8 eV when considering the conduction band offsets between Si-H-Si and $(SiH)_2$, which is in reasonable agreement with the electron energy barrier of ~ 0.6 eV measured in the HRS.[54] The SET transition is initiated when trap-assisted tunneling through the $(SiH)_2$ defect stimulates H desorption to form Si-H-Si.

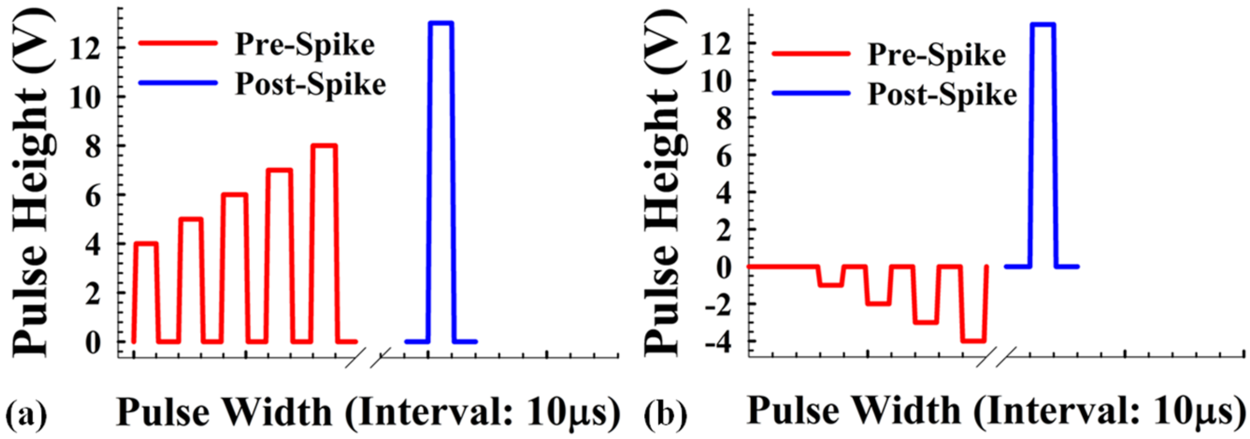

Figure S6. A pulse waveform design using the non-identical pulse method for demonstration of spike-timing-dependent plasticity (STDP) with fixed 10 μs spike pulse width intervals. (**a**) Potentiation of conductance strength change is achieved by using multi-step spike heights from 4 V to 8 V in the pre-neuron state and a single spike height also fixed at 13 V in the post-neuron state. Similarly, depression of conductance strength change can be achieved in (**b**) by using multi-step spike heights from -4 V to 0 V in the pre-neuron state and a single spike height fixed at 13 V in the post-neuron state.
